# Live *Staphylococcus aureus* Induces Expression and Release of Vascular Endothelial Growth Factor in Terminally Differentiated Mouse Mast Cells

**DOI:** 10.3389/fimmu.2016.00247

**Published:** 2016-06-23

**Authors:** Carl-Fredrik Johnzon, Elin Rönnberg, Bengt Guss, Gunnar Pejler

**Affiliations:** ^1^Department of Anatomy, Physiology and Biochemistry, Swedish University of Agricultural Sciences, Uppsala, Sweden; ^2^Department of Medical Biochemistry and Microbiology, Uppsala University, Uppsala, Sweden; ^3^Department of Biomedical Science and Veterinary Public Health, Swedish University of Agricultural Sciences, Uppsala, Sweden

**Keywords:** mast cells, VEGF family, *Staphylococcus aureus*, NF-κB, peritoneal cavity

## Abstract

Mast cells have been shown to express vascular endothelial growth factor (VEGF), thereby implicating mast cells in pro-angiogenic processes. However, the mechanism of VEGF induction in mast cells and the possible expression of VEGF in fully mature mast cells have not been extensively studied. Here, we report that terminally differentiated peritoneal cell-derived mast cells can be induced to express VEGF in response to challenge with *Staphylococcus aureus*, thus identifying a mast cell–bacteria axis as a novel mechanism leading to VEGF release. Whereas live bacteria produced a robust upregulation of VEGF in mast cells, heat-inactivated bacteria failed to do so, and bacteria-conditioned media did not induce VEGF expression. The induction of VEGF was not critically dependent on direct cell–cell contact between bacteria and mast cells. Hence, these findings suggest that VEGF can be induced by soluble factors released during the co-culture conditions. Neither of a panel of bacterial cell-wall products known to activate toll-like receptor (TLR) signaling promoted VEGF expression in mast cells. In agreement with the latter, VEGF induction occurred independently of Myd88, an adaptor molecule that mediates the downstream events following TLR engagement. The VEGF induction was insensitive to nuclear factor of activated T-cells inhibition but was partly dependent on the nuclear factor kappa light-chain enhancer of activated B cells signaling pathway. Together, these findings identify bacterial challenge as a novel mechanism by which VEGF is induced in mast cells.

## Introduction

Mast cells are tissue-resident cells located at the host–environment interface. They express numerous immune receptors and host a multitude of immunological mediators (1–3). While generally known for their involvement in allergy (4), mast cells have also implicated in numerous additional pathological settings, ranging from defense against bacterial infections (5) to an involvement in malignant processes (6). In the latter context, mast cells have, in particular, been implicated to support angiogenesis, thereby promoting tumor growth and metastasis (7, 8).

In the angiogenic process, vascular endothelial growth factor (VEGF) has a key role by promoting endothelial cell survival, proliferation, and migration (9). Moreover, VEGF has been found to exert a chemoattractant effect on immune cells (10). In support for the notion that mast cells are involved in angiogenesis, human, rat, and murine mast cells have been shown to synthesize and secrete VEGF. Previous work has shown that mast cells can release VEGF in response to IgE receptor cross-linking, through stimulation of c-kit, by challenge with a protein kinase C activator (phorbol myristate acetate) or calcium ionophore (11, 12). Additionally, mast cells have been shown to release VEGF in response to PGE_2_ activation through the EP(2) receptor (13), and the adenosine analog [5′-*N*-ethylcarboxamido adenosine (NECA)] has been reported to increase VEGF expression in human lung mast cells (14).

In a previous study, we studied the impact of *Staphylococcus aureus* (*S. aureus*) on gene expression patterns in mast cells (15). As judged by gene array data, we found that live *S. aureus* induced the expression of numerous pro-inflammatory genes such as various cytokines and chemokines. Somewhat unexpectedly, we also found that VEGF was markedly upregulated. In fact, the gene array experiment indicated that the VEGF gene was induced to a higher extent than most other genes. Since no previous study has suggested a link between bacterial infection and induction of VEGF in mast cells, we, here, investigated the relevance of this finding. Moreover, since most of the previous studies in which mast cells have been shown to express VEGF were performed using relatively immature mast cells, we also sought to investigate whether fully mature mast cells can be induced to express VEGF. Indeed, we here provide evidence that live *S. aureus* induces high levels of VEGF expression in terminally differentiated mast cells. Hence, our findings provide a hitherto unrecognized link between mast cells and VEGF expression in the context of bacterial infection.

## Materials and Methods

### Bacteria and Conditioned Media

*Staphylococcus aureus* (strain 8325-4) was streaked on horse blood agar plates (5%; National Veterinary Institute, Uppsala, Sweden) and incubated at 37°C for 24 h. Liquid cultures were started by inoculating 20 ml of Tryptone Soy Broth (TSB; BD) followed by incubation at 37°C and 150 rpm for 16 h. Two hundred microliters of this overnight culture were used to inoculate 20 ml of fresh TSB followed by incubation at 37°C and 150 rpm to an OD_600_ of 1.0. Conditioned media were produced by culturing *S. aureus* in 20 ml of TSB or antibiotic-free peritoneal cell-derived mast cells (PCMCs) media for 24 h at 37°C. The bacteria were removed by centrifugation (6000 × *g* for 5 min) followed by sterile filtration through 0.2 μm filters. Sterility was checked by plating a 100-μl aliquot onto horse blood agar followed by incubation at 37°C for 24 h.

### Peritoneal Cell-Derived Mast Cells

Peritoneal cell-derived mast cells (PCMCs) were established according to a published protocol (16). Briefly, peritoneal lavage of mice was performed, followed by culture of the peritoneal cells in DMEM plus GlutaMAX (Gibco, Invitrogen, Paisley, UK) supplemented with 10% supernatant of stem cell factor-transfected Chinese hamster ovary cells (a gift from Dr. M. Daeron, Pasteur Institute, France), 10% fetal bovine serum, 50 μg/ml streptomycin, 60 μg/ml penicillin, 10 mM MEM non-essential amino acids, and 50 μM 2-mercaptoethanol. The medium was changed every 3–4 days. The inclusion of stem cell factor in the medium promotes the expansion of mast cells at the expense of other peritoneal cell populations. After ~1 month, pure mast cell cultures were obtained, as judged by toluidine blue staining.

### Mice

Female mice of the C57BL/6 background were used for the experiments. All animal experiments were approved by the local ethical committee (Uppsala djurförsöksetiska nämnd; C31/14).

### *In Vitro* Exposure of PCMCs to *S. aureus*, Bacterial Cell-Wall Components, and Conditioned Media

PCMCs were washed twice in PBS and re-suspended in antibiotic-free PCMC medium and plated in 24-well tissue plates at a density of 0.5 × 10^6^ cells per replicate. Alternatively, PCMCs were plated in Transwell plates (0.4 μm pores; Costar). For inhibition experiments, PCMCs were pretreated with 10 μM nuclear factor of activated T-cells (NFAT) inhibitor (11R-VIVIT; Calbiochem, Darmstadt, Germany), 200 nM nuclear factor kappa light-chain enhancer of activated B cells (NF-κβ) inhibitor [6-amino-4-(4-phenoxyphenylethylamino)quinazoline] for 1 h, or with 45 μM Myd88 inhibitor (Pepinh-MYD and control peptide Pepinh-Control) for 6 h prior to infection. The bacteria were washed twice in PBS and added to the PCMC cultures at a final concentration of ~1.25 × 10^7^ CFU/ml; multiplicity of infection (MOI) 25. For inactivation experiments, the bacteria were heat inactivated (HIA) at 60°C for 1 h. Conditioned media were added at a volume corresponding to that of the added bacteria. Purified bacterial cell-wall components: lipopolysaccharide (LPS; 1 μg/ml), lipoteichoic acid (LTA; 1 μg/ml), peptidoglycan (PGN; 10 μg/ml), or Pam3CSK4 (PAM3; 0.5 μg/ml) were added in some experiments. At various time points after infection, cells were collected by centrifugation (6000 × *g*; 5 min). Media and cell fractions were frozen and stored at −20 and −80°C, respectively. All experiments were performed in quadruplicates.

### RNA Preparation and Quantitative Real-Time PCR

Quantitative real-time PCR (qPCR) was performed as described (17). Briefly, total RNA from the co-culture pellets was isolated using the NucleoSpin RNA II kit (Macherey-Nagel, Düren, Germany). qPCR was performed using the SYBR GreenER qPCR Supermix Universal Mastermix (Invitrogen, Waltham, MA, USA) on an ABI 7900HT Fast Real-Time PCR System (Applied Biosystems). The following primers were used: VEGF forward, 5′-GGAGTCTGTGCTCTGGGATT and VEGF reverse, 5′-AACCAACCTCCTCAAACCGT. HPRT forward, 5′-GATTAGCGATGATGAACCAGGTTA and HPRT reverse, 5′-GACATCTCGAGCAAGTCTTTCAGTC. Melt curve analyses of all qPCR products were performed. Relative expression of the VEGF gene in comparison with the house keeping gene (HPRT) was calculated, as previously described (17).

### ELISAs

ELISAs for murine VEGF (PeproTech) were performed according to the manufacturer’s instructions.

### Statistical Analysis

Statistical analyses were performed using one-way ANOVA without matching and Fisher’s LSD *post hoc* test. The analyses were carried out with GraphPad Prism 6 (GraphPad Software). The results shown are from individual experiments, representative for at least two experiments.

## Results

### *S. aureus* Induces VEGF Expression and Release in Cultured Peritoneal Cell-Derived Mast Cells

**Figure 1:**
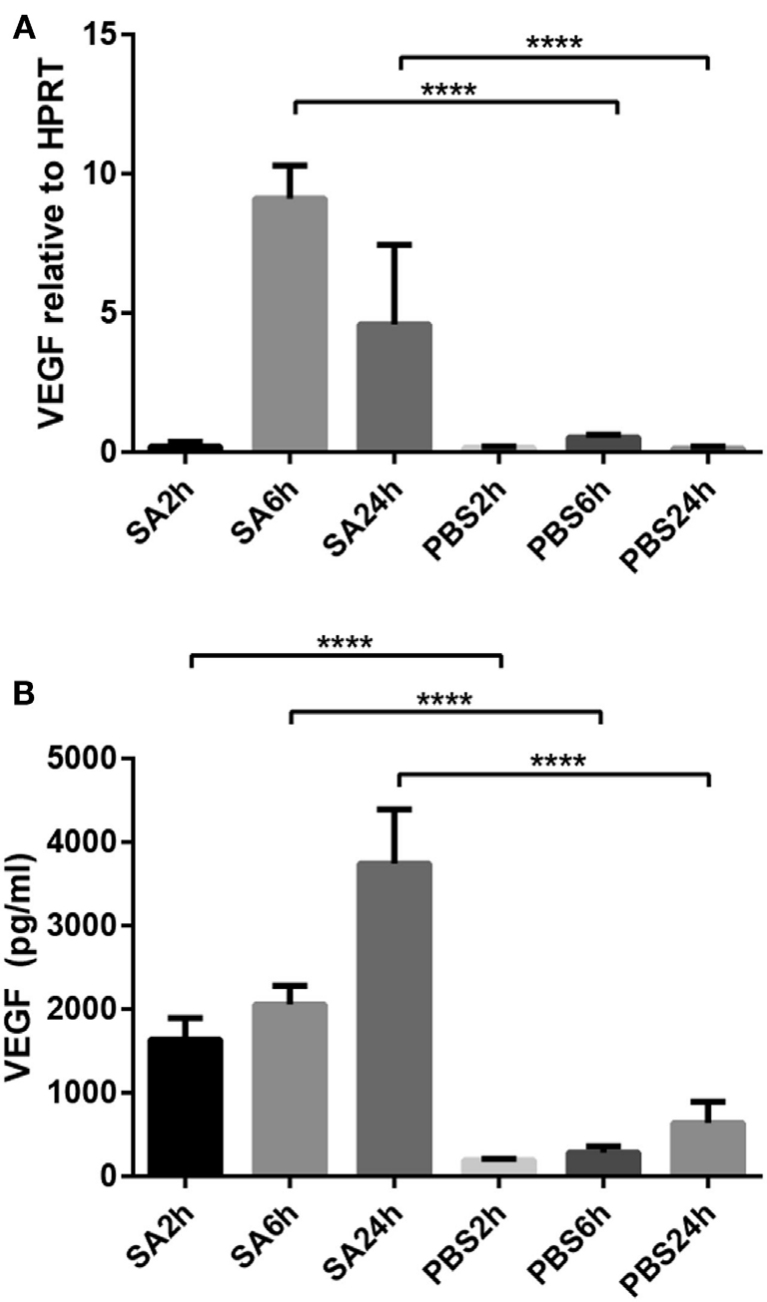
**Co-culture of PCMCs and *S. aureus* induces the expression and release of vascular endothelial growth factor (VEGF)**. Mast cells (PCMCs) were co-cultured with *S. aureus* (SA) with PBS as negative control. Cell fractions were taken at indicated time points, and VEGF expression was measured by qPCR **(A)**. Release of VEGF protein was measured by ELISA **(B)**. Results are given as mean ± SD, ****p < 0.0001 (*n* = 4).

To investigate whether *S. aureus* can affect VEGF expression in mast cells, terminally differentiated peritoneal cell-derived mast cells (PCMCs; 0.5 × 10^6^) were co-cultured with live *S. aureus* (MOI = 25). Cell pellets and supernatants were collected after 2, 6, and 24 h. Total RNA from cell pellets was used for reverse transcription and qPCR. Supernatants were analyzed by ELISA for content of VEGF protein. As shown in Figure 1A, VEGF gene expression was highly induced in the PCMCs after co-culture with the live *S. aureus*. Notably, VEGF expression was modest after 2 h of co-culture and reached a maximum after 6 h.

Increased VEGF gene expression was also accompanied by release of VEGF protein as determined by ELISA. As depicted in Figure 1B, VEGF release was seen from 2 h and onward, with gradually increasing accumulation of VEGF in the medium up to 24 h.

### Live *S. aureus* Induces VEGF Expression in Mast Cells Independent of Bacterial Cell-Wall Components

**Figure 2:**
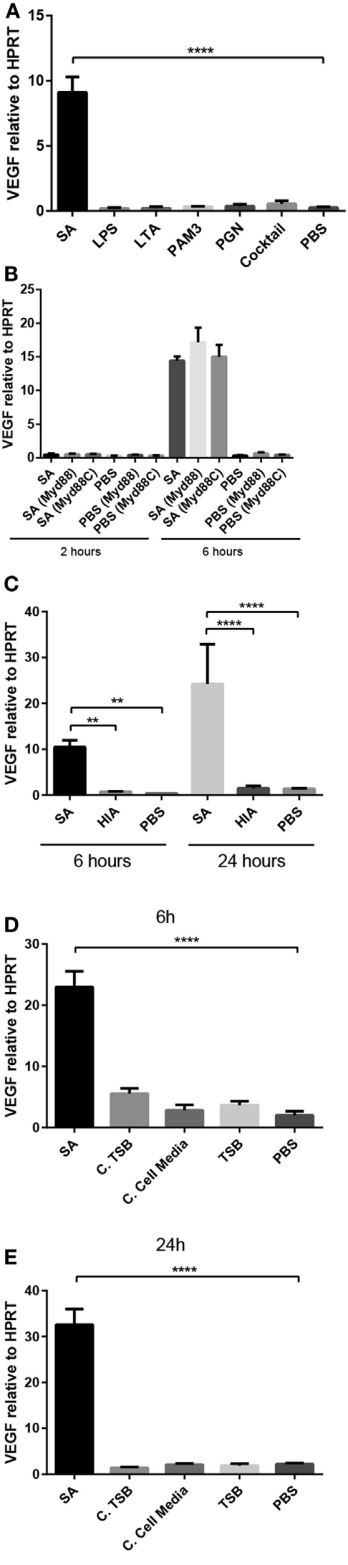
**The induction of VEGF expression in PCMCs requires live bacteria**. **(A)** VEGF mRNA expression in PCMCs in response to bacterial cell-wall components alone or in combination (“Cocktail”): lipopolysaccharide (LPS; 1 μg/ml), lipoteichoic acid (LTA; 1 μg/ml), peptidoglycan (PGN; 10 μg/ml), Pam3CSK4 (PAM3; 0.5 μg/ml); PBS, negative control. **(B)** VEGF mRNA expression in PCMCs preincubated with Myd88 inhibitor (Pepinh-MYD) or with Pepinh-Control (control for the Myd88 inhibitor; Myd88C) followed by co-culture with *S. aureus* (SA) for 2 or 6 h. **(C)** VEGF expression in response to heat-inactivated (HIA) *S. aureus* (SA); PBS, negative control. **(D,E)** VEGF expression in PCMCs at 6 h **(D)** and 24 h **(E)** in response to *S. aureus*-conditioned media. Conditioned media were taken from *S. aureus* cultured in either TSB (bacterial growth medium; C. TSB) or in PCMC medium (C. Cell Media), using TSB or PBS as negative controls. Results are given as mean ± SD, **p < 0.01, and ****p < 0.0001 (*n* = 3–4).

To approach the mechanism by with the bacteria induce VEGF expression in mast cells, we investigated the possibility that VEGF is induced by various toll-like receptor (TLR) ligands that are expressed by bacteria. To this end, PCMCs were stimulated with typical cell-wall components of Gram-positive bacteria: lipoteichoic acid (LTA), PGN, or Pam3CSK4 (PAM3). In addition, we assessed the effect of LPS, i.e., the prototype cell-wall component of Gram-negative bacteria. The bacterial products were added to the mast cells, either alone or in combination, followed by measurement of VEGF gene expression. However, neither of these TLR ligands induced VEGF expression in PCMCs (Figure 2A), suggesting that bacteria induce VEGF by mechanisms in mature mast cells independent of bacterial cell-wall compounds and TLR signaling. In agreement with this notion, inhibition of Myd88, an adaptor molecule common for most TLR signaling pathways, did not reduce the expression of VEGF in response to stimulation of mast cells by live *S. aureus* (Figure 2B).

Next, we assessed whether VEGF induction requires that the bacteria are alive, by investigating the effect of heat-inactivated bacteria on VEGF expression in mast cells. As seen in Figure 2C, HIA *S. aureus* did not induce VEGF expression above baseline levels, indicating that it is essential that the bacteria are alive to be able to induce VEGF expression in mature mast cells. Moreover, since most bacterial cell-wall components are not affected by heat inactivation, this finding further supports that the VEGF induction in mast cells is not mediated by cell-wall components of *S. aureus*.

As VEGF expression was not induced by any of the tested cell-wall compounds, we assessed the possibility that *S. aureus* secrete soluble compounds that might drive VEGF expression. To test this, we collected conditioned media from *S. aureus*, having non-conditioned medium as control. For this experiment, *S. aureus* was either cultured in bacterial growth medium (TSB) or in the medium used for culture of the PCMCs. However, neither of these variants of conditioned media induced the expression of VEGF in PCMCs (Figures 2D,E).

### Optimal Induction of VEGF Expression in Mast Cell Is Dependent of Direct Contact between Mast Cells and Bacteria

**Figure 3:**
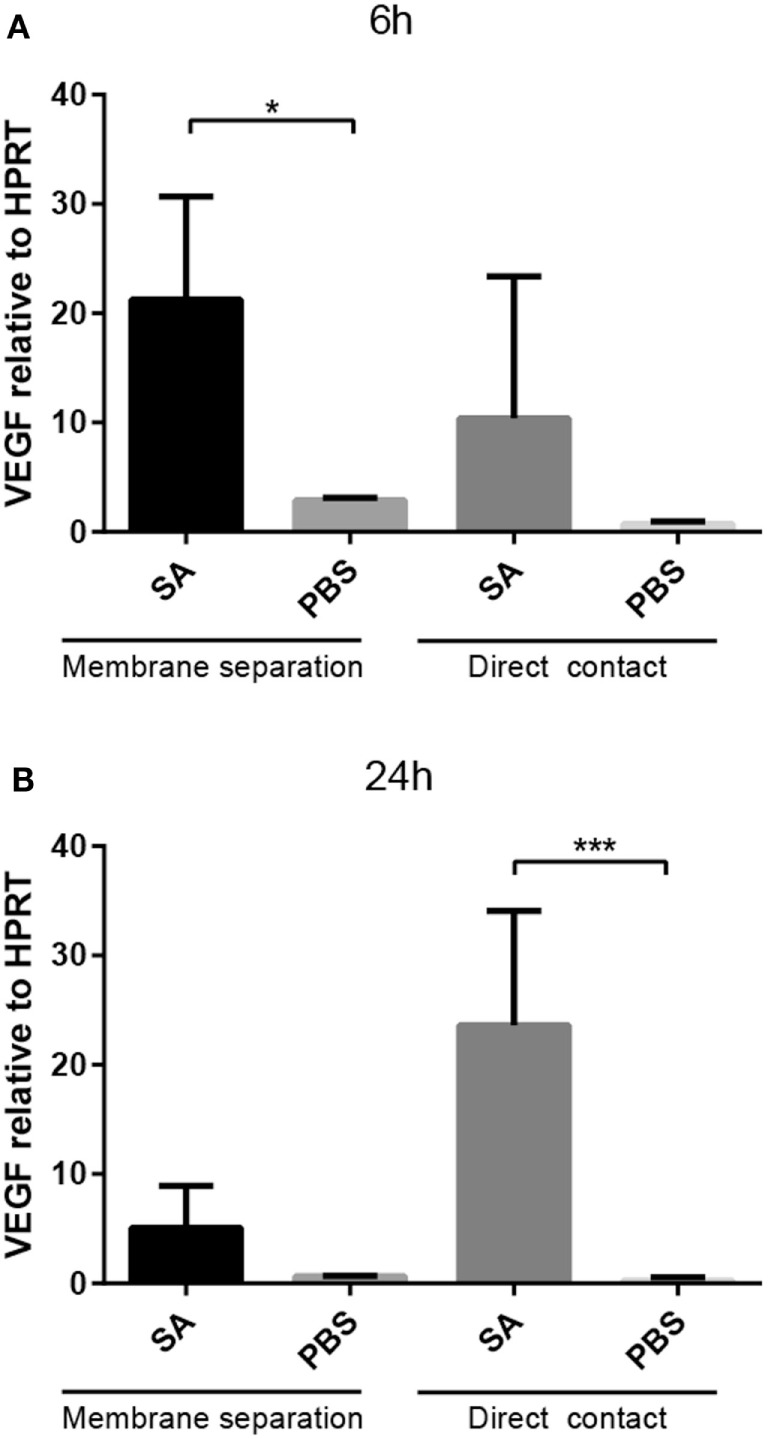
**Upregulation of VEGF does not require direct contact between *S. aureus* and PCMCs**. *Staphylococcus aureus* (SA) was co-cultured for 6 h **(A)** or 24 h **(B)**, either in direct contact or in Transwell conditions as indicated. Cells were recovered and were analyzed for VEGF expression by qPCR. Results are given as mean ± SD, *p < 0.05, and ***p < 0.001 (*n* = 3).

To determine whether the induction of VEGF expression in mast cells by *S. aureus* is dependent on physical contact between the bacteria and mast cells, PCMCs were co-cultured with *S. aureus* in a Transwell system in which the mast cells and bacteria were separated by a 0.4-μm membrane. PCMCs were collected after 6 and 24 h and were assessed for VEGF expression. As shown in Figure 3, the separation of bacteria and mast cells did not obviate the upregulation of VEGF in mast cells at 6 h, suggesting that soluble factors released during co-culture of mast cells and *S. aureus* can account for the induction of VEGF in mast cells. However, it is notable that increased VEGF induction between 6 and 24 h was seen in the direct contact situation, whereas the induction was reduced over time when mast cells and bacteria were separated. This indicates that direct cell–cell contact may be required for sustained VEGF induction at high levels.

### VEGF Upregulation in *S. aureus*-Stimulated Mast Cells Is Independent on NFAT but Partly Dependent on NF-κB

**Figure 4:**
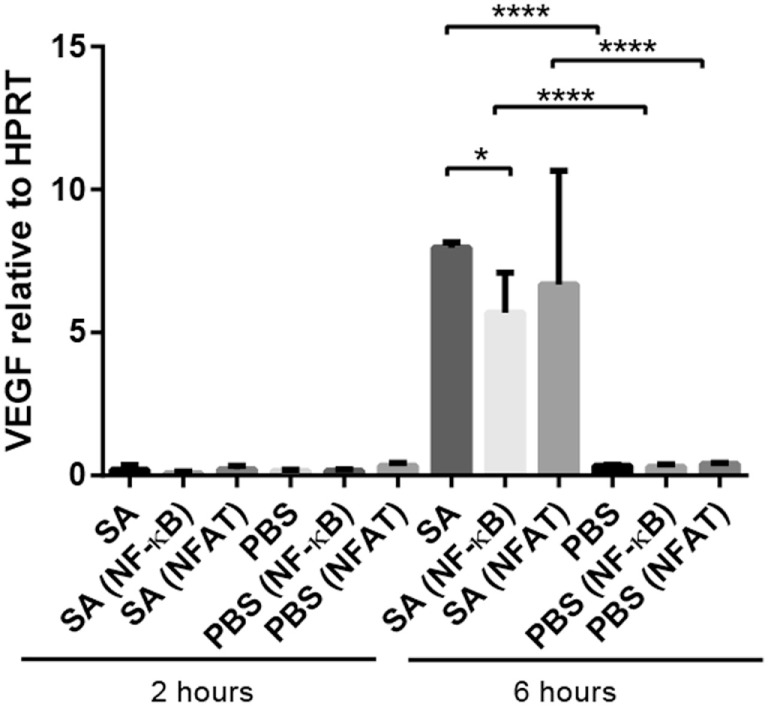
**Effect of NF-κB, and NFAT inhibition on VEGF expression in *S. aureus*-stimulated mast cells**. Mast cells (PCMCs) were preincubated with inhibitors of NFAT (11R-VIVIT) or NF-κB [6-amino-4-(4-phenoxyphenylethylamino)quinazoline] followed by co-culture with *S. aureus* (SA) for 2 or 6 h. VEGF expression was assessed by qPCR. Results are given as mean ± SD, *p < 0.05, and ****p < 0.0001 (*n* = 3–4).

The inhibition of NFAT, a signaling molecule that previously has been shown to have role in the induction of pro-inflammatory genes in mast cells (18, 19), did not significantly affect the induction of VEGF by *S. aureus* (Figure 4). In contrast, NF-κB inhibition produced a modest, yet significant, reduction in VEGF expression in mast cells (Figure 4). Hence, the upregulated VEGF expression in mast cells stimulated with live *S. aureus* is partly dependent on the NF-κB signaling pathway.

## Discussion

Mast cells are emerging as major detrimental effector cells in numerous pathophysiological conditions, not only in allergy but also in diverse processes such as autoimmune disease, atherosclerosis, cancer, obesity, and contact dermatitis (20, 21). On the other hand, mast cells can also be beneficial to their host, as exemplified by the contribution of mast cells to the host response against bacterial insult (1–3). Although the mechanism by which mast cells influence these processes can vary, there is a widespread notion that mast cells are a source of numerous growth factors, such as basic fibroblast growth factor, nerve growth factor, platelet-derived growth factor, and VEGF (21). Among these, the expression of VEGF by mast cells has attracted particular attention because of the implication of mast cells in malignant processes, where mast cells are thought to promote tumor angiogenesis by secreting growth factors including VEGF (22–25). However, although mast cells are emerging as major VEGF-producing cells, there is still limited knowledge of the mechanisms of VEGF induction in mast cells.

Here, we report that mast cells can be induced to express high levels of VEGF in response to bacterial insult, thus introducing a bacteria–mast cell axis as a mechanism for production of this growth factor. It is also important to stress that most previous studies in which mast cells were shown to express VEGF were focused on relatively immature mast cells, whereas we here report VEGF expression by fully mature mast cells. Interestingly, we noted a robust release of VEGF already after 2 h, whereas the onset of VEGF gene expression occurred at a later stage. This indicates that the early VEGF secretion is due to release of preformed VEGF from stores in granules, whereas the induction of VEGF gene expression may serve to maintain high levels of VEGF release after the preformed stores have been emptied.

The expression of VEGF in response to bacterial challenge could potentially have various pathophysiological consequences. One obvious scenario could be that mast cell-derived VEGF could have a role in the angiogenesis that accompanies the wound healing process following a bacterial infection. Alternatively, mast cell-expressed VEGF could promote vascular permeability and leukocyte attraction, thereby contributing to the primary host response following a bacterial insult. On a different angle, it is possible that VEGF expression induced in mast cells by bacteria, in fact, could be of relevance for the progress of malignant processes. Several species of bacteria and bacterial strains are known to populate tumors. These bacteria may either be the cause of the tumor or may represent an opportunistic infection occurring as a consequence of the immunosuppressed status of the tumor tissue (26). It is also well known that mast cells populate a wide range of tumors, often being located at the tumor periphery but also within the actual tumor (7, 8). Possibly, bacteria populating the tumor may thus cause mast cells to upregulate their expression of VEGF, and the mast cell-derived VEGF could then have a pathogenic impact by promoting tumor angiogenesis.

Our findings suggest that the induction of VEGF is critically dependent on the interaction of mast cells with live bacteria, whereas various isolated bacterial cell-wall components and heat-inactivated bacteria were without effect. These findings are somewhat surprising considering that mast cells express several TLRs, and that stimulation of these by various PAMPs have previously been shown to induce the expression of pro-inflammatory cytokines in mast cells (27–32). This suggests that the induction of VEGF occurs independently of TLR stimulation and, in support of this, we did not see any effect of an Myd88 inhibitor on VEGF expression in response to bacterial challenge. It was also noted that VEGF induction was not critically dependent on direct cell–cell contact between the bacteria and mast cells, suggesting that VEGF can to some extent be induced by soluble factors released by the bacteria. Intriguingly though, such factors were not found in conditioned medium obtained by culturing *S. aureus* alone, either in bacterial growth medium or in the medium used for culture of the mast cells. Hence, the release of factors promoting VEGF expression in mast cells appears to require communication between live bacteria and mast cells, leading to the induction and release of VEGF-driving soluble factors. Although we are at present not able to specify the nature of such factors, we noted that the induction of VEGF expression in mast cells was partly dependent on the NF-κB pathway. Altogether, the present findings suggest that soluble, VEGF-driving factors are released by live *S. aureus* as a consequence of crosstalk between live *S. aureus* and mast cells.

## Author Contributions

C-FJ planned and performed most of the experimental work, interpreted data, and wrote the manuscript; ER performed experimental work, interpreted data, contributed to the planning of the study, and to the writing of the paper; BG contributed to the experiments and to the design of the study; GP planned the study, interpreted data, and wrote the manuscript.

## Conflict of Interest Statement

The authors declare that the research was conducted in the absence of any commercial or financial relationships that could be construed as a potential conflict of interest.
